# Optimising Management of Paediatric Distal Radius Torus Fractures: A Complete Two-Cycle Audit Assessing Compliance With National Institute for Health and Care Excellence (NICE) Guideline NG38

**DOI:** 10.7759/cureus.109442

**Published:** 2026-05-22

**Authors:** Praveen Rajan, Srinath Pammi, Venkata Nutalapati, Yousef El-Tawil, Bhargava Krishna Balineni, Ilias Seferiadis

**Affiliations:** 1 Trauma and Orthopaedics, Basildon University Hospital, Basildon, GBR; 2 Trauma and Orthopaedics, Mid and South Essex NHS Trust, Basildon, GBR

**Keywords:** buckle fracture, clinical audit, distal radius, fracture clinic, immobilisation, nice ng38, paediatric, quality improvement, torus fracture

## Abstract

Background: Distal radius torus (buckle) fractures are among the most common paediatric injuries presenting to emergency departments. National Institute for Health and Care Excellence (NICE) guideline NG38 recommends management with non-rigid immobilisation and discharge without routine follow-up. Despite robust evidence supporting this approach, clinical practice varies considerably across NHS settings.

Aim: This study aims to assess baseline compliance with NICE NG38 in the management of paediatric distal radius torus fractures, implement targeted quality-improvement interventions, and evaluate their effect through a complete closed-loop audit cycle.

Methods: A retrospective two-cycle clinical audit was conducted at a District General Hospital (DGH) and its associated Minor Injury Unit (MIU) within the same NHS trust in the United Kingdom. Cycle 1 (February 2023 to August 2023, n = 222) established baseline practice. Interventions included clinician education, guideline dissemination, and visual prompts. Cycle 2 (March 2024 to September 2024, n = 258) evaluated post-intervention practice. Data were collected from electronic emergency department records, radiographic systems, and Virtual Fracture Clinic (VFC) documentation.

Results: In Cycle 1, compliance with NICE NG38 was 97/174 (56%) at the DGH and 0/48 (0%) at the MIU; 128/222 (58%) of all patients across both sites received unnecessary fracture clinic follow-up. Following interventions, Cycle 2 demonstrated improved compliance to 131/164 (80%) at the DGH and 29/94 (31%) at the MIU. Overall fracture clinic follow-up was reduced from 128/222 (58%) to 98/258 (38%). Rigid immobilisation persisted in 33/164 (20%) of DGH patients and 65/94 (69%) of MIU patients in Cycle 2.

Conclusion: This closed-loop audit demonstrates that targeted educational and environmental interventions can meaningfully improve adherence to evidence-based guidelines and reduce unnecessary fracture clinic referrals. However, variability in immobilisation practice persists, particularly at the MIU, underscoring the need for sustained system-level interventions, standardised clinical pathways, and ongoing audit cycles.

## Introduction

Distal radius torus (buckle) fractures are the most frequent skeletal injuries in children, accounting for a substantial proportion of paediatric emergency department attendances in the United Kingdom and worldwide [1,2]. These are stable low-energy compression injuries of the cortex in which the trabecular bone remains intact, conferring minimal risk of displacement and an excellent prognosis with conservative management [3,4].

The landmark FORCE (Forearm Fracture Recovery in Children Evaluation) randomised controlled trial demonstrated that rigid plaster immobilisation provides no statistically or clinically significant advantage over removable splinting in terms of pain, functional recovery, or complication rates [1]. Multiple earlier trials and systematic reviews had already confirmed equivalent outcomes with reduced healthcare utilisation when non-rigid management is employed [5-7].

On the basis of this evidence, the National Institute for Health and Care Excellence (NICE) guideline NG38 [8] recommends no rigid cast, use of a removable splint or supportive bandage, and discharge from the emergency department without routine orthopaedic follow-up. Despite this clear national guidance, studies consistently demonstrate ongoing overuse of rigid immobilisation and unnecessary follow-up appointments across NHS trusts, representing avoidable patient burden and resource inefficiency [9-11]. Implementation of evidence-based guidelines into routine clinical practice remains a recognised challenge in emergency and orthopaedic settings.

This paper presents a complete two-cycle closed-loop clinical audit conducted at a DGH (District General Hospital) and its associated MIU. The primary objective was to assess baseline compliance with NICE NG38, defined as the proportion of eligible patients managed with non-rigid immobilisation and discharged without routine fracture clinic follow-up. Secondary objectives were to characterise immobilisation type and follow-up rates at each site, implement targeted quality-improvement interventions, and evaluate the impact of those interventions through re-audit.

## Materials and methods

Study design

A retrospective, two-cycle closed-loop clinical audit was conducted in accordance with NHS clinical audit standards. Ethical approval was not required as this was a service evaluation; local audit registration was obtained.

Setting

The audit was conducted at two sites within the same NHS trust in the United Kingdom: a DGH with a full emergency department and on-site orthopaedic services, and an associated Minor Injury Unit (MIU) staffed primarily by emergency nurse practitioners.

Study population

All patients aged under 16 years presenting with an isolated distal radius torus fracture confirmed on plain radiographs during the study period were eligible for inclusion. Patients with associated injuries, open fractures, or fractures requiring operative management were excluded.

Data collection

Data were extracted retrospectively from electronic emergency department records, radiographic reporting systems, the Pathpoint fracture clinic database, and Virtual Fracture Clinic (VFC) documentation. Cases were identified by searching the electronic records system for all paediatric attendances (age under 16 years) with a recorded diagnosis or radiological report containing the terms “torus”, “buckle”, or “distal radius fracture” during the relevant audit periods. Radiological confirmation was reviewed by the lead auditor; cases reported as “angled buckle fractures” or where the report described features of both torus and greenstick morphology were included if the treating clinician documented management as a torus fracture and no operative intervention was performed, as these represent a recognised diagnostic grey zone in which NG38 guidance is commonly applied. Data extraction was performed independently by two members of the audit team, with discrepancies resolved by consensus. Where documentation of immobilisation type or follow-up arrangement was absent or ambiguous, the case was recorded as non-compliant with the relevant component to avoid overestimation of adherence rates. The proportion of cases with missing or unrecordable data for each variable was negligible (fewer than 2% of records per variable) and did not materially affect the results.

Outcome measures

The primary outcome was overall compliance with NICE NG38, defined as treatment with non-rigid immobilisation and discharge without routine fracture clinic follow-up. For the purpose of this audit, “non-rigid immobilisation” was defined as the application of a removable splint (futura-type or equivalent) or a supportive bandage only, without any form of rigid plaster backslab or circumferential cast. “Routine follow-up” was defined as any scheduled attendance at a fracture clinic (FC), Virtual Fracture Clinic (VFC) review, or GP orthopaedic review arranged at the point of ED or MIU discharge, where such follow-up would not have been clinically indicated for an uncomplicated torus fracture. Composite compliance requires both components to be met simultaneously. Secondary outcomes were: type of immobilisation applied (rigid backslab versus removable splint or supportive bandage) and rate of fracture clinic follow-up.

Audit standard

The audit standard was defined as 100% compliance with NICE NG38, in line with the guideline’s specific recommendation that all eligible patients with a distal radius torus fracture should receive: (i) non-rigid immobilisation (removable splint or supportive bandage; no rigid plaster cast) and (ii) discharge from the emergency department without routine orthopaedic follow-up. This two-component standard mirrors the exact NG38 wording and serves as the basis for the composite compliance measure used throughout this audit. A 100% target was adopted because NG38 does not specify clinical exceptions for uncomplicated torus fractures; however, it is acknowledged that clinicians may exercise discretion in atypical presentations (e.g., diagnostic uncertainty, angled buckle pattern, or safeguarding concerns), and such cases were not excluded from the denominator unless they met the pre-specified exclusion criteria (associated injuries, open fractures, or fractures requiring operative management).

Audit cycles

Cycle 1 (Baseline) ran from February 2023 to August 2023 and assessed current practice without prior intervention. Following analysis of Cycle 1 results, the following quality-improvement measures were implemented between October 2023 and February 2024: educational posters summarising the NG38 recommendations were displayed in the ED resuscitation area, minors, and MIU triage bay; NICE NG38 was disseminated to all clinical staff at both sites via departmental email; dedicated teaching sessions (two sessions at DGH, one session at MIU) were delivered by the lead audit clinician to junior doctors, nursing staff, and nurse practitioners, covering the evidence base, guideline recommendations, and correct use of removable splints; and best practice was reinforced at departmental governance meetings. Cycle 2 (Re-audit) then ran from March 2024 to September 2024 to evaluate practice following those interventions.

Statistical analysis

Data were analysed descriptively using frequencies and proportions. All percentage values are accompanied by absolute numbers in N(%) format. Differences in proportions between audit cycles were assessed using the chi-squared (χ²) test, with a two-sided p-value of <0.05 considered statistically significant. Analysis was performed using Microsoft Excel (Microsoft Corporation, Redmond, Washington) and IBM SPSS Statistics for Windows, Version 28 (Released 2021; IBM Corp., Armonk, New York).

## Results

Patient demographics

Cycle 1 included 222 patients (DGH: n = 174; MIU: n = 48). Cycle 2 included 258 patients (DGH: n = 164; MIU: n = 94). The increase in MIU patient numbers in Cycle 2 is consistent with increased service activity over the re-audit period.

Compliance with NICE NG38

In Cycle 1, overall NICE NG38 compliance was 97/174 (56%) at DGH and 0/48 (0%) at MIU, giving a combined cross-site compliance of 97/222 (44%). Following the quality-improvement interventions, compliance improved to 131/164 (80%) at DGH (χ² = 14.2, p < 0.001) and 29/94 (31%) at MIU (χ² = 29.6, p < 0.001), with a combined compliance of 160/258 (62%) in Cycle 2. Table 1 summarises all key outcomes across both audit cycles and sites, including chi-squared statistics and p-values.

**Table 1:** Summary of Key Audit Outcomes by Site and Cycle † Follow-up rate reported across both sites combined; χ² and p-value reflect combined site comparison. * Statistically significant (p < 0.05). DGH: District General Hospital; MIU: Minor Injury Unit; χ² : chi-squared test statistic.

Outcome	Cycle 1 DGH (n = 174)	Cycle 1 MIU (n = 48)	Cycle 2 DGH (n = 164)	Cycle 2 MIU (n = 94)	χ² DGH	p DGH	χ² MIU	p MIU
NICE NG38 compliance	97/174 (56%)	0/48 (0%)	131/164 (80%)	29/94 (31%)	14.2	<0.001*	29.6	<0.001*
Rigid immobilisation	77/174 (44%)	48/48 (100%)	33/164 (20%)	65/94 (69%)	9.8	0.002*	10.4	0.001*
Removable splint use	97/174 (56%)	0/48 (0%)	131/164 (80%)	29/94 (31%)	9.8	0.002*	10.4	0.001*
Fracture clinic follow-up†	128/222 (58%)	—	98/258 (38%)	—	12.1†	<0.001*†	—	—

Immobilisation practices

**Figure 1:**
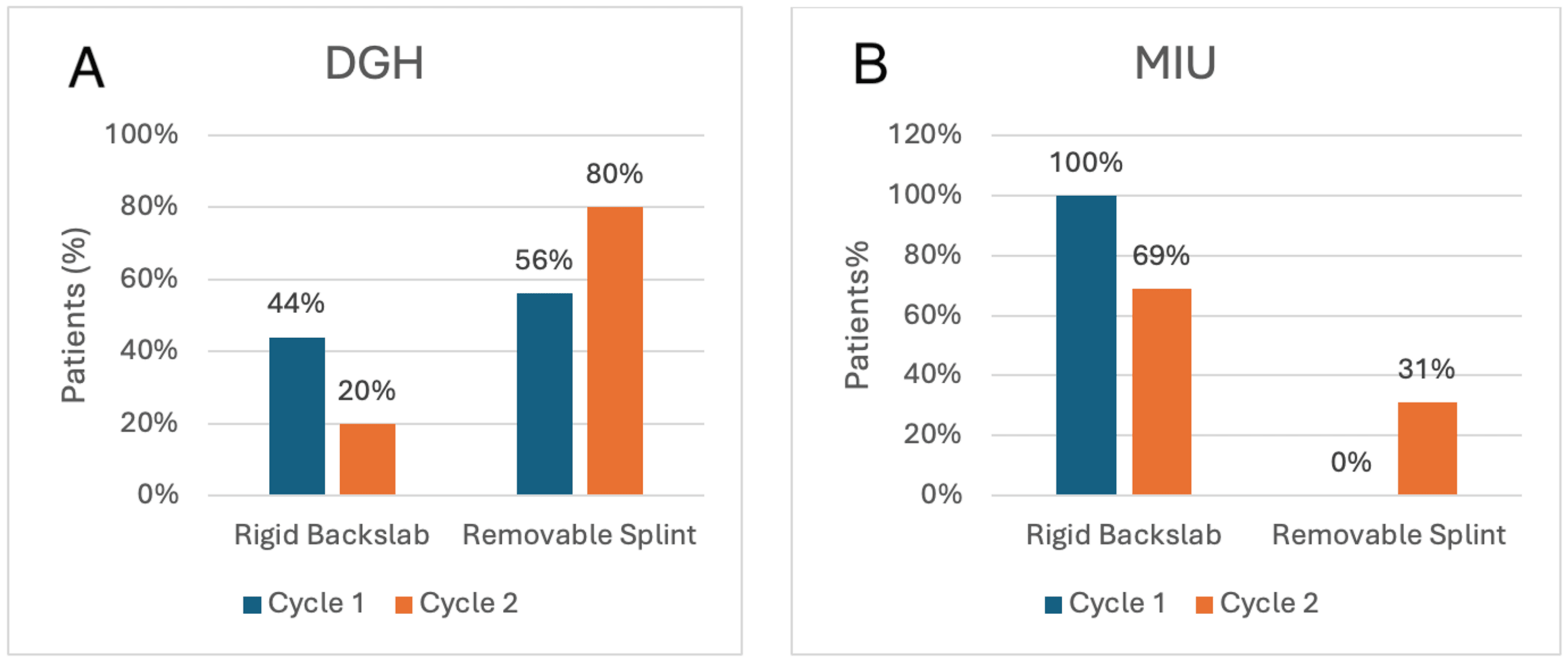
Immobilisation type (rigid backslab vs. removable splint) by site and audit cycle. Panel A: District General Hospital (DGH). Panel B: Minor Injury Unit (MIU). Blue bars represent Cycle 1 (baseline); orange bars represent Cycle 2 (re-audit). Percentages represent the proportion of patients receiving each immobilisation type.

In Cycle 1, 77/174 (44%) of DGH patients and 48/48 (100%) of MIU patients received rigid plaster immobilisation. In Cycle 2, rigid immobilisation was reduced to 33/164 (20%) at DGH and 65/94 (69%) at MIU, representing a statistically significant improvement at both sites (DGH: χ² = 9.8, p = 0.002; MIU: χ² = 10.4, p = 0.001). Correspondingly, use of removable splints increased from 97/174 (56%) to 131/164 (80%) at DGH and from 0/48 (0%) to 29/94 (31%) at MIU. Figure 1 illustrates these changes.

Fracture clinic follow-up

**Figure 2:**
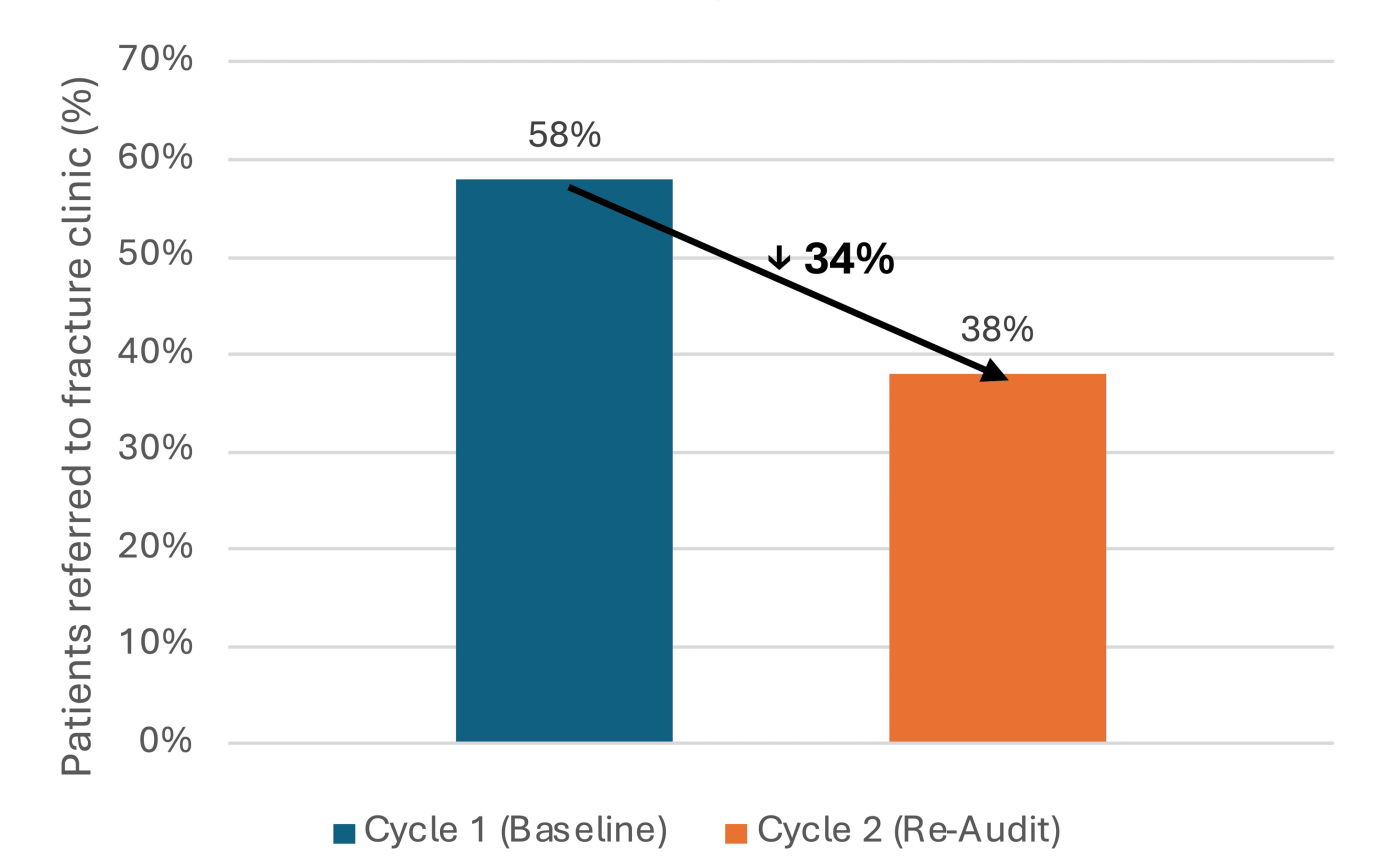
Fracture clinic follow-up rates across audit cycles (combined DGH and MIU). DGH: District General Hospital; MIU: Minor Injury Unit.

In Cycle 1, 128/222 (58%) of patients across both sites were referred to the fracture clinic for follow-up. In Cycle 2, this was reduced to 98/258 (38%) overall, representing a 34% relative reduction (χ² = 12.1, p < 0.001). Follow-up is reported as a combined cross-site figure because within this trust, all fracture clinic appointments, whether the patient initially presented to the ED or the MIU, are conducted at the DGH fracture clinic. The follow-up rate, therefore, reflects a single, shared downstream service rather than two independent pathways, and disaggregation by initial presentation site would not meaningfully alter the interpretation. Figure 2 illustrates this change.

## Discussion

This two-cycle closed-loop audit evaluates the real-world implementation of NICE guideline NG38 for paediatric distal radius torus fractures and demonstrates the measurable impact of targeted quality-improvement interventions. The findings contribute to a growing body of literature documenting persistent variation in clinical practice and the challenges of translating evidence-based guidelines into routine care.

Baseline practice and variation

The first audit cycle revealed substantial deviation from NICE NG38 recommendations. Rigid immobilisation was applied in 77/174 (44%) of DGH patients and 48/48 (100%) of MIU patients, and 128/222 (58%) of all patients were referred for fracture clinic follow-up despite evidence that such follow-up confers no clinical benefit [1,3]. The complete non-compliance at MIU at baseline likely reflects the absence of site-specific guideline implementation at that unit prior to the audit. These findings are consistent with national and international reports of persistent over-treatment of torus fractures despite the availability of high-quality evidence and clear national guidance [9]. The discrepancy between guideline publication and clinical implementation is a well-recognised challenge in healthcare quality improvement.

Impact of interventions

Following targeted interventions, such as clinician education, guideline dissemination, and environmental prompts, significant improvements were observed across both sites. Compliance at DGH improved from 97/174 (56%) to 131/164 (80%), and at MIU from 0/48 (0%) to 29/94 (31%). The reduction in fracture clinic follow-up from 128/222 (58%) to 98/258 (38%) represents a meaningful improvement in service efficiency, releasing clinic capacity for patients who genuinely require orthopaedic review. The marked improvement at MIU from zero baseline compliance is noteworthy and aligns with evidence that settings with the greatest deviation from best practice may demonstrate the largest relative response to structured interventions [5,7]. However, compliance at both sites remains below the 100% audit standard, indicating the need for further work.

Barriers to guideline implementation

Persistent variation in immobilisation practice may reflect a range of organisational and behavioural barriers. The persistently high rate of rigid immobilisation at the MIU specifically warrants consideration: while clinician habit and preference for familiar practice are likely contributors, it is also possible that stock availability of removable splints at the MIU played a role, as this unit did not consistently carry the same range of splint sizes as the DGH. The audit methodology did not formally distinguish between cases of non-compliance attributable to splint unavailability versus those reflecting clinician choice; this distinction has implications for the type of system-level intervention required and should be explored in future quality-improvement work. More broadly, clinician habit, experience, and preference for rigid immobilisation remain significant drivers of non-compliance [12-14], compounded by high staff turnover, particularly among junior clinicians rotating through emergency settings. Variable availability of and familiarity with removable splints, perceived medicolegal risk associated with minimal intervention [15,16], and insufficient embedding of guideline teaching into induction programmes all contribute. These factors are consistent with the implementation science literature, which identifies organisational, cultural, and resource-related determinants as central challenges to guideline adherence [11,13,14].

Clinical and service implications

The most clinically impactful finding is the reduction in unnecessary fracture clinic attendances. For a condition that is prevalent and generally self-limiting, avoidance of unnecessary follow-up reduces patient burden, frees orthopaedic capacity for complex cases, and reduces associated healthcare costs. At the population level, given the high incidence of torus fractures in children, even modest improvements in guideline adherence can translate to substantial system-wide savings [16]. Moreover, following NICE NG38 reduces clinic visits, radiographs, and costs while improving patient satisfaction [15,17].

Strengths

This study has several methodological strengths. It presents a complete closed-loop audit cycle-essential for quality improvement-with a prospectively defined standard (100% compliance with NICE NG38). The combined sample size of 480 patients across two sites provides sufficient statistical power to detect meaningful differences between cycles. Data were obtained from multiple electronic systems, reducing the risk of ascertainment bias.

Limitations

Several limitations should be acknowledged. The retrospective design may introduce documentation bias, as the treatment rationale was not always recorded. The study was conducted within a single NHS trust, which may limit generalisability to other settings. Patient-reported outcomes, including pain scores, functional recovery, and satisfaction, were not collected, precluding evaluation of clinical effectiveness. The chi-squared analyses assume independence between cycles, which is appropriate given non-overlapping patient cohorts; however, potential confounders, such as case-mix variation across periods, were not formally adjusted for. No “balancing measures” (such as reattendance rates, complication rates, or unplanned re-presentations) were collected; future audit cycles should incorporate these to ensure that improvements in guideline adherence are not associated with unintended adverse outcomes. Intervention fidelity was not formally assessed: it is possible that educational sessions were attended by varying proportions of clinical staff across sites, and that poster visibility and awareness of guideline updates differed between the DGH and MIU. This limits the ability to attribute observed improvements to specific intervention components with certainty.

Future directions

Sustained improvement will require system-level change beyond periodic education. Development and implementation of standardised clinical pathways for torus fracture management, integration of electronic clinical decision-support tools within emergency department systems, and consistent availability of removable splints at both DGH and MIU are recommended. Embedding NICE NG38 training into junior doctor and nurse practitioner induction programmes, and establishing a regular audit-and-feedback cycle to sustain improvements over time, are also essential.

## Conclusions

This complete two-cycle closed-loop audit demonstrates that targeted, multimodal quality-improvement interventions can produce measurable and clinically significant improvements in adherence to NICE NG38 for paediatric distal radius torus fractures. Simple, low-cost interventions such as clinician education, guideline dissemination, and environmental prompts can shift practice at both a specialist emergency department and a nurse-led minor injury unit, though achieving 100% compliance will require system-level change: standardised clinical pathways, electronic decision-support, consistent availability of removable splints, and embedding guideline training into staff induction programmes. Given the high prevalence of torus fractures in the paediatric population, full implementation of NICE NG38 across NHS trusts represents a significant opportunity to improve patient experience, reduce unnecessary clinical contacts, and release orthopaedic capacity, outcomes that are achievable, measurable, and aligned with patients’ best interests.
